# Supplementary material for: Constituents of Aquilaria sinensis Leaves Upregulate the Expression of Matrix Metalloproteases 2 and 9
Source: Molecules. 2021 Apr 26;26(9):2537. doi: 10.3390/molecules26092537 (PMC8123650; doi:10.3390/molecules26092537)
Supplement: Supplementary file 1 [file molecules-26-02537-s001.zip › molecules-1171307-supplementary.pdf]

molecules

Supplementary Materials

Constituents of *Aquilaria sinensis* Leaves Upregulate the Expressions of Matrix Metalloproteases 2 and 9

Sui-Wen Hsiao[1,†], Yu-Chin Wu[2,†], Hui-Ching Mei[3], Yu-Hsin Chen[4], George Hsiao[5]* and Ching-Kuo Lee[1,2,6,7]*

[1] Ph.D. Program in Drug Discovery and Development Industry, Taipei Medical University, 250 Wu Xin Street, Taipei 11031, Taiwan; suifeng0506@gmail.com
[2] Graduate Institute of Pharmacognosy, Taipei Medical University, 250 Wu Xin Street, Taipei 11031, Taiwan; tmc761038@tmu.edu.tw
[3] Department of Science Education, National Taipei University of Education, Taipei 10671, Taiwan; hcmei@tea.ntue.edu.tw
[4] Taichung District Agricultural Research and Extension Station, Council of Agriculture, Executive Yuan, Taichung 42081, Taiwan; ychen@tdais.gov.tw
[5] Department of Pharmacology, Taipei Medical University, 250 Wu Xin Street, Taipei 110, Taiwan; geohsiao@tmu.edu.tw
[6] School of Pharmacy, Taipei Medical University, 250 Wu Xin Street, Taipei 110, Taiwan; cklee@tmu.edu.tw
[7] Department of Chemistry, Chung Yuan Christian University, Zhongbei Road, Zhongli District, Taoyuan City 320314, Taiwan
* Correspondence: cklee@tmu.edu.tw, Tel.: +886 2 27361661 #6150
† Equal contribution with the first author

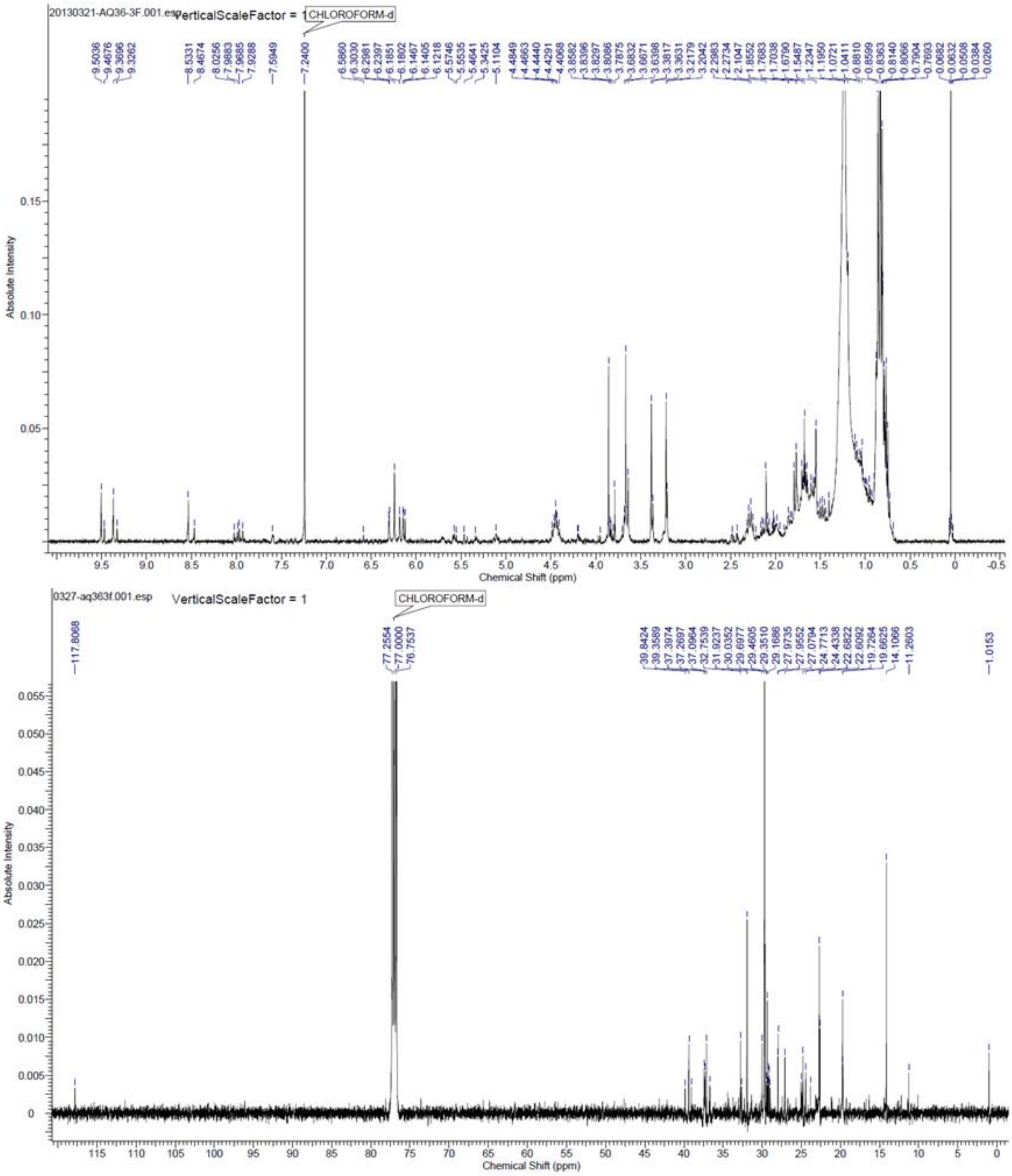

Figure S1. NMR spectrum of AQ20. ^{1}H NMR spectrum (300 MHz, CD_3OD) and ^{13}C NMR spectrum (125 MHz, CD_3OD) of AQ20.

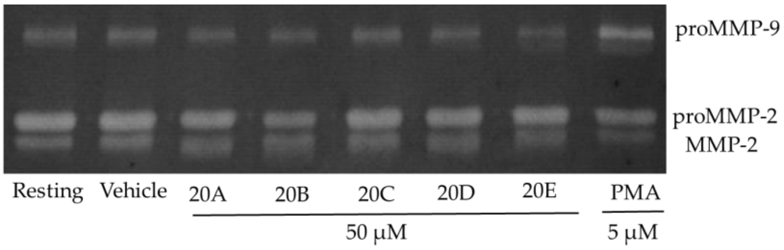

Figure S2. Effects of the five PA analogues on MMP-2 and MMP-9 expression
